# The Bone–Brain Axis: Novel Insights into the Bidirectional Crosstalk in Depression and Osteoporosis

**DOI:** 10.3390/biom16020213

**Published:** 2026-01-31

**Authors:** Pengpeng Li, Yangyang Gao, Xudong Zhao

**Affiliations:** 1 Department of Neurosurgery, Northwest University Affiliated Xi’an Aerospace Hospital, Yanta District, Xi’an 710000, China; 2 Department of Neurosurgery, School of Medicine, Ningxia Medical University, Yinchuan 750004, China; 3 Wuxi School of Medicine, Jiangnan University, Wuxi 214122, China

**Keywords:** bone–brain axis, depression, osteoporosis, osteocalcin, lipocalin-2, neuroinflammation

## Abstract

Depression and osteoporosis frequently co-occur, presenting a significant and increasing clinical challenge, especially among older adults. Growing research highlights the bone–brain axis, a complex bidirectional communication network connecting the skeletal and central nervous systems, as a central mechanism linking these conditions. This review comprehensively examines the current knowledge of the molecular and cellular pathways within this axis that contribute to depression–osteoporosis interactions. It details how depression promotes bone loss through sustained hypothalamic–pituitary–adrenal axis activation, sympathetic nervous system overactivity, and chronic low-grade inflammation. This review also explores how bone-derived factors, including osteocalcin, lipocalin 2, and extracellular vesicles, cross the blood–brain barrier to influence brain function by regulating hippocampal neurogenesis, serotonin signaling, and neuroinflammation. This bidirectional communication is modulated by circadian rhythms and genetic factors. Understanding these pathways offers critical insights into the shared pathophysiology and reveals promising therapeutic targets. Interventions such as neuromodulation, customized exercise programs, and novel treatments focusing on bone-derived signals show potential for simultaneously addressing both mood disorders and bone health deterioration. This review emphasizes the need for an integrated system-based approach in clinical care that moves beyond traditional specialty-focused treatment to improve overall health outcomes, particularly for vulnerable elderly individuals.

## 1. Introduction

### 1.1. Depression as a Global Health Challenge

Depression, a complex neuropsychiatric disorder, arises from the intricate interplay of genetic predisposition, environmental stressors, and neurobiological alterations, and it is emerging as one of the most significant public health challenges of the 21st century [1]. According to the World Health Organization (WHO), depression affects over 280 million individuals worldwide and ranks among the leading causes of disability and mortality, with its disease burden continuing to escalate [2]. Projections from the WHO indicate that, by 2030, depression will become the primary contributor to the global disease burden [2]. Notably, the COVID-19 pandemic exacerbated this public health crisis. The 2020 Global Burden of Disease Study revealed a 27.6% increase in the incidence of major depressive disorder (MDD) across 204 countries and regions due to pandemic-related measures [3]. Despite available treatments including pharmacological, psychotherapeutic, and neuromodulation approaches, approximately one-third of patients with severe depression remain inadequately responsive [4], underscoring the urgent need for novel therapeutic strategies and deeper mechanistic insights.

### 1.2. The Skeletal System as an Endocrine Organ and the Emergence of the Bone–Brain Axis

Bones were traditionally viewed as primarily for mechanical support and mineral homeostasis, but they are now recognized as dynamic endocrine organs [5]. The emerging evidence suggests that bones and the brain interact through multiple mechanisms to influence the onset and progression of depression. However, systematic reviews in this field remain lacking.

This recognition has been propelled by advances in inter-organ communication research, revealing a complex bidirectional interaction between the skeletal and central nervous systems. This interplay forms the basis of the innovative “bone–brain axis” concept [6]. Bones, therefore, are not merely passive structural entities but highly active endocrine organs [7], secreting various bioactive factors—such as osteopontin, osteocalcin, lipocalin-2, and fibroblast growth factors—that can remotely modulate brain function through endocrine pathways [8].

Conversely, the brain regulates bone homeostasis and regeneration via efferent neural pathways [9]. It influences bone metabolism through multiple mechanisms, including the hypothalamic–pituitary axis, the sympathetic nervous system, and neuropeptide release [10]. The discovery of this bidirectional crosstalk provides a novel theoretical framework for understanding the comorbidity between depression and bone-related disorders, such as osteoporosis.

**Figure 1:**
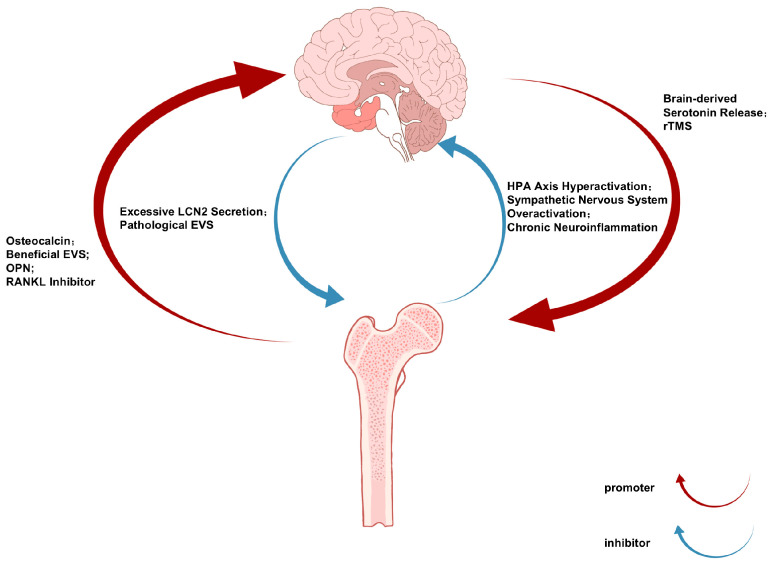
The Bone-Brain Communication Axis in Depression and Osteoporosis: Bidirectional Signaling Pathways and Therapeutic Implications.

The bidirectional communication of the bone–brain axis operates through a sophisticated neuroendocrine–immune network, with the brain regulating bone metabolism via three principal pathways [11]: (1) hypothalamic-mediated sympathetic activation releases norepinephrine to suppress osteoblast activity and promote osteoclast differentiation through β2-adrenergic receptor signaling [12]; (2) anterior pituitary hormones including growth hormones and thyroid-stimulating hormones modulate bone remodeling through endocrine actions [13]; and (3) there is direct neural regulation via sensory fiber-derived neuropeptides such as CGRP and substance P [14]. Conversely, skeletal-derived factors including osteocalcin, lipocalin-2, and sclerostin influence CNS function by crossing the blood–brain barrier or activating vagal afferents, thereby regulating neurogenic processes, synaptic plasticity, and neurotransmitter homeostasis [15]. This reciprocal crosstalk establishes an integrated physiological framework connecting skeletal and neural pathophysiology (Figure 1).

Notably, certain bone-derived factors and cells can cross the blood–brain barrier to directly influence the central nervous system [16,17]. Under neuropsychiatric conditions, compromised blood–brain barrier integrity may facilitate the entry of these osteogenic substances into brain tissue, where they exert regulatory effects. Psychological stress has been shown to promote myelopoiesis and exacerbate neuroinflammation through the brain–bone marrow axis, potentially triggering depressive symptoms [18].

### 1.3. Clinical and Epidemiological Evidence Linking Depression and Osteoporosis

The bone–brain axis plays a pivotal regulatory role in osteoarthritis pathogenesis through integrated neuronal pathways, molecular signaling, circadian rhythms, and genetic factors [19]. This paradigm highlights the skeletal system’s underrecognized contribution to neuropsychiatric disorders, particularly depression. Supporting this connection, clinical observations reveal that depression patients frequently present with comorbid skeletal disorders such as osteoporosis [20], suggesting the bone–brain axis may mediate this bidirectional relationship.

Clinical epidemiological studies provide compelling evidence supporting the bone–brain axis theory. Substantial research demonstrates that patients with depression frequently exhibit skeletal health impairments, including reduced bone mineral density and increased fracture risk. A cross-sectional study involving 4719 participants (depressed patients versus healthy controls) revealed significantly lower bone density and elevated fracture incidence among the depression cohort [21]. Conversely, osteoporosis patients demonstrate higher prevalence rates of depressive symptoms [22]. This bidirectional association suggests shared pathophysiological mechanisms between depression and osteoporosis, with the bone–brain axis potentially serving as a critical interface mediating this comorbidity [23]. Recent reviews have further expanded the understanding of the broad role of this axis in both physiological and pathological states, highlighting its centrality in connecting neuropsychiatric disorders and metabolic bone diseases [24].

### 1.4. Purpose and Scope of This Review

Building upon the established clinical association and the emerging mechanistic framework of the bone–brain axis, this review aims to provide a focused synthesis of current knowledge specifically linking depression and osteoporosis. While the broader roles of the axis are being recognized [24], a detailed examination dedicated to this high-impact comorbidity is warranted. Herein, we will systematically detail the mechanistic pathways underlying the “brain-to-bone” and “bone-to-brain” communication relevant to depression and osteoporosis, evaluate key clinical and translational evidence, and discuss the resulting implications for novel therapeutic strategies. Our goal is to consolidate existing insights and highlight future directions for research and the integrated clinical management of these co-occurring conditions.

## 2. Mechanisms Underlying Depression-Mediated Skeletal Impairment

Depression exerts detrimental effects on skeletal health through intricate biological and behavioral mechanisms that collectively form a multidimensional pathological network [25]. From a biological perspective, chronic hyperactivity of the hypothalamic–pituitary–adrenal (HPA) axis represents the central pathway mediating depression-induced bone loss [26].

Under chronic stress conditions, the paraventricular nucleus of the hypothalamus increases secretion of the corticotropin-releasing hormone (CRH), which stimulates the anterior pituitary release of the adrenocorticotropic hormone (ACTH), ultimately leading to excessive glucocorticoid (primarily cortisol) production by the adrenal cortex [27]. These persistently high glucocorticoid levels exert multifaceted detrimental effects on bone homeostasis, including direct suppression of osteoblast differentiation and proliferation, induction of osteoblast and osteocyte apoptosis, and enhancement of osteoclastogenesis and activation, thereby creating an imbalanced bone remodeling state, characterized by reduced bone formation and increased resorption [28].

Clinical observations further support this mechanism, demonstrating that depressed patients exhibit significantly higher 24-h urinary free cortisol excretion compared to healthy controls, with cortisol levels showing an inverse correlation with bone mineral density [29,30], highlighting the critical role of HPA axis dysregulation in depression-associated osteoporosis.

Hyperactivation of the sympathetic nervous system (SNS) represents another critical mechanism linking depression to skeletal deterioration [12]. Genetic studies in murine models demonstrate that SNS dysfunction or deficiency promotes bone mass accumulation [31], suggesting its regulatory role in bone homeostasis. Catecholamines released during sympathetic activation exert dual detrimental effects on bone metabolism through β2-adrenergic receptors on osteocytes: (1) suppression of the Wnt/β-catenin signaling pathway (a master regulator of osteogenesis) and (2) upregulation of receptor activator of nuclear factor kappa-B ligand (RANKL) expression (the pivotal mediator of osteoclast differentiation). These molecular events collectively establish an imbalanced bone remodeling state characterized by attenuated formation and enhanced resorption [32].

At the molecular level, dysregulated inflammatory networks constitute a shared pathological foundation connecting depression and osteoporosis [33,34]. Clinical investigations consistently report elevated proinflammatory cytokines (including IL-6, TNF-α, and IL-1β) in depressed patients [35,36,37], which also participate in osteoporotic pathogenesis through multiple mechanisms: (1) redirecting differentiation of bone marrow mesenchymal stem cells toward adipocytes rather than osteoblasts and (2) activating and maturing osteoclast precursors [38,39]. Genome-wide pleiotropic analyses have identified 65 genetic loci jointly associated with both skeletal degenerative diseases and mental disorders, with the IL6R and TNF gene loci showing particular prominence. The significant enrichment of these genes in neural tissues and immune pathways strongly implicates neuroinflammatory responses as the molecular bridge connecting depression and bone loss [40].

Notably, Mendelian randomization analyses reveal differential associations between mental disorders and skeletal pathologies: major depressive disorder (MDD) increases the risk for intervertebral disc degeneration (IVDD), while schizophrenia (SCZ) may conversely reduce the knee osteoarthritis risk (Table 1). This disorder-specific association pattern suggests distinct mechanistic pathways through which various psychiatric conditions influence skeletal health [40].

Behavioral factors play a significant role in depression-associated bone loss [41]. Patients with depression frequently exhibit behavioral alterations including reduced physical activity, vitamin D deficiency due to insufficient sunlight exposure, and eating disorders, all of which can directly or indirectly impair bone metabolism [42]. Moreover, the higher prevalence of smoking and alcohol abuse among depressed individuals not only exacerbates the depressive symptoms but also further compromises skeletal health [43].

Of particular note, certain antidepressant medications (especially selective serotonin reuptake inhibitors, SSRIs) may exert direct effects on bone metabolism [44]. Research indicates that long-term SSRI use increases fracture risk, potentially mediated through the widespread expression of serotonin receptors in bone tissue [45]. However, clinical studies examining the impact of antidepressants on bone mineral density have yielded inconsistent results, likely attributable to variations in the drug class, dosage, and duration of treatment [46]. In contrast to the complex effects of pharmacological modulation, the emerging evidence suggests that enhancing endogenous brain-derived serotonin signaling through specific pathways may offer a beneficial strategy. For instance, a recent study demonstrated that diosgenin alleviated postmenopausal bone loss by activating ERα-dependent PI3K/AKT/GSK3β signaling to enhance brain-derived serotonin, providing a novel mechanistic insight into the ‘brain-to-bone’ protective pathway [47].

To provide a clearer overview of the evidence supporting these pathways, Table 2 categorizes the key studies discussed in this section based on their mechanistic focus and study design.

## 3. The Regulatory Effects of the Skeletal System on Depression

Emerging research has fundamentally transformed the traditional view of the skeletal system as a passive target of neural regulation, instead revealing its active role as an endocrine organ capable of the profound modulation of brain function [48]. Contemporary studies demonstrate that bone tissue participates in depression pathophysiology through three principal mechanisms: (1) secretion of osteokines (particularly osteocalcin), (2) extracellular vesicle-mediated interorgan communication, and (3) the modulation of the bone marrow microenvironment [25]. These paradigm-shifting discoveries not only provide novel insights into depression pathogenesis but also identify promising therapeutic targets for next-generation antidepressants.

Emerging evidence has established osteocalcin as a crucial endocrine mediator in bone–brain crosstalk [49], with its uncarboxylated form (ucOC) demonstrating the ability to cross the blood–brain barrier and modulate neural function [50]. Preclinical studies reveal that peripheral osteocalcin administration alleviates depression-like behaviors in animal models while upregulating hippocampal BDNF expression and promoting neurogenesis [51]. The molecular mechanism involves osteocalcin binding to GPR158 receptors on GABAergic neurons, thereby suppressing their inhibitory control over serotonergic neurons in the dorsal raphe nucleus and ultimately enhancing prefrontal serotoninergic neurotransmission, a pathway remarkably similar to conventional antidepressant action [51,52]. Clinically, depressed patients exhibit significantly lower serum osteocalcin levels that inversely correlate with the symptom severity [53], collectively suggesting this osteokine serves as an endogenous mood regulator. These findings not only elucidate a novel endocrine axis connecting skeletal and mental health but also identify osteocalcin signaling as a potential therapeutic target for mood disorders.

Osteopontin (OPN) represents another crucial hormonal mediator in bone–brain communication, initially identified for its role in osteoblast adhesion [54]. This multifunctional protein exerts significant effects through several mechanisms: (1) modulating the balance between pro- and anti-inflammatory responses, (2) maintaining blood–brain barrier integrity, (3) inhibiting cellular apoptosis, and (4) promoting neuronal migration and proliferation—collectively contributing to tissue remodeling and functional recovery [8]. Genetic studies have revealed associations between OPN gene polymorphisms and depression susceptibility [55]; however, the precise mechanisms underlying OPN’s potential role in depression pathophysiology require further investigation [56].

Extracellular vesicles (EVs) have emerged as novel mediators in bone–brain communication, capable of transporting bioactive molecules including microRNAs (miRNAs), long non-coding RNAs (lncRNAs), and proteins across organ systems [57,58]. Pioneering work by Professor Lu Shen and Professor Hui Xie’s team at Xiangya Hospital, Central South University, has significantly advanced this field. Their research demonstrated that brain-derived EVs from Alzheimer’s disease (AD) mouse models were enriched with miR-483-5p, which could traverse the blood–brain barrier to reach bone tissue. These EVs suppressed osteogenic differentiation, while promoting the adipogenic differentiation of bone marrow mesenchymal stem cells (BMSCs) through the downregulation of insulin-like growth factor 2 (Igf2) expression, ultimately leading to osteoporosis [57].

The team previously discovered that EVs derived from young osteocytes could enter the brain and improve cognitive function in AD mice, revealing the bidirectional nature of bone–brain crosstalk [58]. These collective findings illuminate the critical role of EV-mediated interorgan communication in both neurological and skeletal disorders, providing novel insights into the shared pathophysiology between depression and osteoporosis (Table 3).

RANKL (Receptor Activator of Nuclear Factor κB Ligand), secreted primarily by osteoblasts and osteocytes, serves as a critical regulator of bone metabolism under both physiological and pathological conditions [59]. Emerging evidence reveals its dual role in neuropsychiatric disorders, where it modulates neuroinflammatory responses in depression. Notably, anti-RANKL therapy has demonstrated therapeutic potential by simultaneously ameliorating depressive behaviors and improving bone metabolic abnormalities [60], suggesting a shared mechanistic pathway between skeletal and mood disorders.

Emerging research has identified lipocalin-2 (LCN2) as a pivotal signaling molecule in the bone–brain axis that critically regulates various neuropsychiatric disorders [61]. Clinical investigations reveal that serum LCN2 levels in acute ischemic stroke (AIS) patients at admission show a significant positive correlation with the post-stroke depression (PSD) risk [62]. A prospective cohort study by Liu et al. involving 358 AIS patients demonstrated that elevated serum LCN2 levels remained an independent risk factor for PSD development even after adjusting for age, stroke severity, and baseline depressive symptoms, suggesting LCN2’s potential as an early predictive biomarker for PSD [62].

Mechanistic studies using animal models have elucidated multiple pathways through which LCN2 participates in mood regulation [63]. Under chronic stress conditions, wild-type mice exhibit characteristic depression-like phenotypes including reduced hippocampal neurogenesis, anxiety-like behaviors, and memory dysfunction, whereas LCN2 knockout mice show remarkable resistance to these behavioral abnormalities [64]. Notably, mouse models of inflammatory bowel disease display significantly upregulated brain LCN2 expression accompanied by distinct depression-like behaviors, both of which can be effectively ameliorated through either genetic knockout or neutralizing antibody blockade of LCN2 function [63]. These collective findings strongly indicate that LCN2 likely contributes to depression pathogenesis across various disease states by impairing hippocampal neuroplasticity.

Circadian rhythm disruption has emerged as a pivotal interface connecting skeletal and neuropsychiatric pathophysiology through bidirectional regulatory mechanisms [19]. The coordinated expression of core clock genes (Clock, Bmal1, Period, and Cryptochrome) in both osseous and neural tissues orchestrates not only local physiological processes but also systemic interorgan communication via endocrine and neural pathways [65]. Clinical observations reveal a striking comorbidity of circadian disturbances, with osteoporotic patients demonstrating altered sleep architecture and melatonin secretion profiles that mirror the characteristic circadian phase abnormalities observed in major depressive disorder [66,67]. Of particular pathophysiological significance, osteoarthritis-induced dysregulation of peripheral circadian oscillators initiates a cascade of events, including modified osteokine secretion patterns and proinflammatory signaling, that propagate through the bone–brain axis to disrupt central pacemaker function, thereby establishing a self-perpetuating vicious cycle [19]. This mechanistic understanding of circadian coupling between skeletal and neural systems provides a novel conceptual framework for explaining psychiatric–skeletal comorbidity while simultaneously identifying chronobiological interventions (e.g., targeted melatonin receptor agonists or timed light therapy) as promising therapeutic strategies capable of concurrently addressing both mood disturbances and metabolic bone disease.

## 4. Clinical Translation Prospects of Bone–Brain Axis Research

The establishment of the bone–brain axis theory not only provides a new framework for understanding the comorbidity mechanism between depression and osteoporosis but also opens innovative pathways for developing novel intervention strategies. Based on the current research findings, multimodal interventions targeting the bone–brain axis may become an important direction for future clinical practice, encompassing drug development, neuromodulation technologies, exercise prescription, and nutritional interventions. These innovative strategies are expected to break through the limitations of traditional single-disease treatment and achieve the synergistic management of both depression and osteoporosis.

Non-invasive brain stimulation techniques have emerged as promising therapeutic tools for modulating the bone–brain axis function [68], with transcranial magnetic stimulation (TMS) and transcranial direct current stimulation (tDCS) demonstrating particular efficacy in clinical applications [69]. These neuromodulation approaches appear to exert dual beneficial effects, as evidenced by studies showing that high-frequency repetitive TMS applied to the prefrontal cortex not only alleviates depressive symptoms but also elevates serum markers of bone formation, suggesting a coordinated improvement in both neurological and skeletal parameters through bone–brain axis modulation [70]. The safety profile and minimal side effects of these techniques make them especially suitable for elderly patients with comorbid conditions, although the optimal stimulation parameters and long-term outcomes require further investigation [19]. Current research efforts are focusing on elucidating target-specific effects of different cortical stimulation sites (e.g., motor cortex versus prefrontal cortex) on bone metabolism, while also exploring potential synergistic effects when combined with pharmacological interventions, representing important directions for advancing this novel treatment paradigm.

Exercise intervention represents one of the most promising non-pharmacological approaches for simultaneously improving depressive symptoms and skeletal health [71,72]. Physical activity modulates the bone–brain axis function through multiple mechanisms: acute exercise stimulates hypothalamic–pituitary–adrenal (HPA) axis and sympathetic nervous system activity, while chronic exercise enhances the stress adaptation capacity of these systems, thereby mitigating skeletal damage from chronic stress [73]. Research demonstrates that exercise increases the secretion of insulin-like growth factor 1 (IGF-1), which not only promotes osteoblast proliferation and differentiation but also crosses the blood–brain barrier to act on the prefrontal cortex and hippocampus, enhancing neuroplasticity and antidepressant effects [74,75]. The significant correlation between exercise-mediated antidepressant effects and bone quality improvement suggests bone-derived factors (e.g., osteocalcin) are key mediators of these dual benefits [76]. Optimization of exercise prescriptions for different populations (e.g., adolescents, menopausal women, and the elderly) remains an important research direction, requiring comprehensive consideration of the exercise type, intensity, frequency, and duration for their specific effects on the bone–brain axis.

The psychiatric effects of bone-targeting medications represent another promising research frontier [6]. Growing attention has focused on the neurocognitive impacts of conventional anti-osteoporotic agents, including bisphosphonates, RANKL inhibitors, and parathyroid hormone (PTH) analogs [77]. Clinical observations reveal that teriparatide (PTH1-34) treatment not only improves bone mineral density but also alleviates depressive symptoms in osteoporotic patients, an effect potentially mediated through upregulated brain-derived neurotrophic factor (BDNF) expression [78]. Similarly, denosumab, a RANKL inhibitor, has demonstrated mood-enhancing effects in animal models alongside its established skeletal benefits [60].

Although preliminary, these findings suggest certain bone-modifying drugs may confer mental health advantages through bone–brain axis modulation. Rigorous randomized controlled trials are needed to verify these clinical observations and elucidate the underlying molecular mechanisms. Of particular therapeutic interest is the development of novel osteokine modulators, such as osteocalcin analogs and LCN2-neutralizing antibodies, which hold unique potential for concurrently addressing both skeletal and psychiatric disorders through targeted endocrine pathways.

Emerging evidence highlights circadian rhythm modulation as a promising therapeutic strategy for the bone–brain axis, given the coordinated operation of autonomous clock systems in both skeletal and neural tissues that critically maintain systemic homeostasis [79,80]. Clinical observations demonstrate that osteoarthritis patients with circadian disruptions exhibit concurrent benefits in both joint pathology and mood states when treated with chronotherapeutic interventions such as timed light exposure or melatonin supplementation [81]. Mechanistic insights from preclinical models reveal that the osteoblast-specific deletion of the core clock gene Bmal1 induces parallel anxiety-like behaviors and bone loss, suggesting skeletal-derived circadian signals may directly influence neural function [82]. These findings carry particular translational significance for vulnerable populations such as shift workers, elderly individuals, and postmenopausal women who experience disproportionate circadian disruption while exhibiting elevated comorbid risks for depression and osteoporosis, making them ideal candidates for targeted chronotherapeutic interventions that may simultaneously address both skeletal and psychiatric health through bone–brain axis modulation (Table 4).

## 5. Discussion

### 5.1. Integrative Framework and Clinical Significance

This review establishes the bone–brain axis as a fundamental physiological framework that mechanistically links two major age-related conditions: depression and osteoporosis. By synthesizing the evidence from molecular pathways, clinical observations, and therapeutic studies, we demonstrate that this relationship represents a bidirectional causal dialogue mediated through neuroendocrine, inflammatory, and osteokine signaling pathways. The clinical significance of this integrated perspective lies in its potential to transform the management of these comorbid conditions, shifting from siloed specialty care toward holistic interventions that address mental and skeletal health concurrently.

### 5.2. Critical Analysis of the Mechanistic Evidence

When examining the brain to bone pathways, the evidence shows remarkable consistency across different study types. Clinical findings of HPA axis hyperactivity [29,30] align well with experimental models demonstrating glucocorticoid-induced bone loss [28], while genetic studies further support shared inflammatory pathways [40]. However, certain complexities emerge upon closer examination. For instance, while sympathetic activation consistently shows detrimental skeletal effects in experimental models [31,32], human studies using beta blockers present more nuanced outcomes, suggesting possible compensatory mechanisms or population-specific factors.

The bone to brain evidence reveals even more complexity. Osteocalcin demonstrates compelling preclinical evidence for antidepressant effects through GPR158-mediated serotonergic modulation [50], yet translational human data remain limited to correlational studies [51]. Conversely, lipocalin 2 presents a paradox [59]. While human studies associate elevated levels with post stroke depression, animal models show both neuroinflammatory [60] and potentially context-dependent effects. This contrast highlights the importance of distinguishing between acute pathological and chronic physiological signaling contexts.

Therapeutic studies offer particularly insightful comparisons. While SSRIs show concerning associations with fracture risk [45], neuromodulation approaches such as rTMS demonstrate potential dual benefits [67], suggesting that the method of neuronal intervention critically determines the skeletal outcomes. Similarly, the contrast between the skeletal risks of pharmacological serotonin modulation versus the protective effects of exercise-enhanced osteocalcin signaling [73] underscores that pathway specificity within the bone–brain axis determines the therapeutic success.

Recent research reveals additional dimensions to this pharmacological interplay. Investigations into the cholinergic system, which has not been traditionally emphasized in depression pathophysiology, demonstrate that acetylcholinesterase inhibitors commonly prescribed for cognitive impairment also influence bone remodeling processes. These agents appear to modulate osteoclast differentiation and activity through mechanisms involving both neural and peripheral pathways [83]. This finding extends the conceptual boundaries of bone–brain communication, suggesting that pharmacological interventions targeting neurological function may exert concurrent effects on skeletal integrity. Future work should clarify whether such mechanisms contribute to the clinical relationship between neuropsychiatric disorders and bone health, potentially revealing novel targets for dual therapeutic approaches.

### 5.3. Methodological Considerations and Future Directions

Several methodological factors must be considered when interpreting this evidence. First, most osteokine research remains preclinical, with human studies primarily correlational. Second, the temporal dynamics of bone–brain communication, particularly in chronic conditions such as depression, are poorly understood. Third, significant sex and age differences in both depression and osteoporosis pathophysiology suggest the bone–brain axis may operate differently across populations, though current studies often fail to stratify by these variables.

Future research should prioritize several key areas: longitudinal human studies measuring both osteokine profiles and neuropsychiatric outcomes; advanced imaging techniques to visualize bone–brain communication in vivo; mechanistic studies examining circadian influences on axis function; and targeted clinical trials of axis-modifying interventions in carefully phenotyped populations. The recent identification of shared genetic loci offers promising avenues for precision medicine approaches, particularly if combined with dynamic biomarkers of axis activity.

From a clinical translation perspective, the most immediate applications may involve integrating existing axis-sensitive interventions, such as exercise protocols and circadian rhythm optimization, into standard care for both conditions. More targeted approaches, including osteocalcin-based therapeutics or LCN2 modulation, require further validation but represent promising frontier interventions.

### 5.4. Limitations and Concluding Synthesis

This review has several limitations. The relatively nascent state of bone–brain axis research means many proposed mechanisms lack extensive replication. The publication bias toward positive findings may overestimate certain effects. Additionally, our focus on depression and osteoporosis necessarily excludes discussion of the axis’s roles in other neurological or skeletal conditions.

Nevertheless, the convergence of evidence across multiple disciplines strongly supports the bone–brain axis as a legitimate and clinically relevant paradigm. The bidirectional mechanisms we have delineated provide not only explanatory power for epidemiological observations but also a roadmap for developing novel therapeutic strategies. As research in this field advances, we anticipate that interventions targeting this axis will increasingly move toward clinical application, ultimately improving outcomes for the millions affected by these interconnected conditions.

## 6. Conclusions

This review has systematically addressed a central scientific question of how the bone–brain axis functionally links two prevalent age-related disorders, depression and osteoporosis. By synthesizing molecular, clinical, and translational research, we demonstrate that this axis represents a legitimate physiological network rather than a speculative construct.

We have characterized two interdependent pathological processes. On the one hand, depression induces skeletal deterioration through chronic overactivity of the stress-responsive hypothalamic–pituitary–adrenal axis, excessive sympathetic signaling via β-adrenergic pathways, and a sustained pro-inflammatory state. On the other hand, bone-derived factors actively participate in neuropsychiatric regulation. Uncarboxylated osteocalcin penetrates the blood–brain barrier to enhance neurogenesis and serotonin signaling, while lipocalin-2 contributes to neuroinflammatory cascades. These reciprocal communications are further modulated by circadian oscillators and genetic predispositions.

The clinical implications are substantial and immediate. Our findings advocate for a paradigm shift from compartmentalized treatment toward integrated care models. Promising therapeutic strategies include neuromodulation techniques that may dually benefit mood and bone metabolism, structured exercise programs that stimulate osteokine-mediated neuroprotection, and novel pharmacological approaches targeting bone-derived signaling molecules. Chronobiological interventions also warrant consideration as adjunctive therapies.

Collectively, this evidence establishes the bone–brain axis as a critical interface in depression–osteoporosis comorbidity. Clinicians across relevant specialties should recognize the interconnected pathophysiology of these conditions. Future investigations must validate axis-targeted interventions through rigorous clinical trials, but the current knowledge already supports incorporating this conceptual framework into patient management strategies. By acknowledging the fundamental connections between mental and skeletal health, we can develop more comprehensive approaches to improve outcomes for vulnerable populations.

## Figures and Tables

**Table 1:** Principal mechanisms underlying depression-mediated skeletal pathophysiology.

Mechanism Category	Critical Effectors/Pathways	Skeletal Effects	Clinical Evidence
HPA axis activation	CRH, ACTH, cortisol	Inhibits osteoblast differentiation and promotes osteocyte apoptosis	Negative correlation between urinary cortisol and BMD in MDD patients
Sympathetic overactivity	Norepinephrine, β2-AR	Suppresses Wnt signaling and enhances RANKL expression	β-blockers demonstrate protective effects against stress-induced bone loss
5-HT system dysregulation	Peripheral/CNS 5-HT	Peripheral 5-HT inhibits osteogenesis, and CNS 5-HT promotes bone formation	Controversial data on SSRIs’ skeletal effects

**Table 2:** Summary of key evidence for depression-mediated skeletal impairment mechanisms.

Mechanism Pathway	Key Findings	Study Type	Representative References
HPA Axis Activation	Elevated cortisol inhibits osteoblast function and promotes osteoclastogenesis	Experimental/Preclinical	[28]
	Negative correlation between urinary cortisol and BMD in MDD patients	Clinical Study	[29,30]
Sympathetic Overactivity	Norepinephrine via β2-AR suppresses Wnt/β-catenin and enhances RANKL	Experimental/Preclinical	[12,32]
	Genetic SNS deficiency promotes bone mass accumulation (murine models)	Genetic/Experimental Study	[31]
Chronic Inflammation	Pro-inflammatory cytokines (IL-6, TNF-α, IL-1β) are elevated in depression	Clinical Study	[35,36,37]
	Cytokines redirect MSC differentiation, activate osteoclast precursors	Experimental/Preclinical	[38,39]
	Genetic loci (IL6R, TNF) are associated with both depression and skeletal diseases	Genetic Study	[40]
Behavioral Factors	Reduced physical activity, vitamin D deficiency, and eating disorders	Clinical/Observational	[41,42]
	Smoking and alcohol abuse are higher in depression	Clinical/Observational	[43]
Pharmacological Effects	Long-term SSRI use increases fracture risk	Clinical Study	[44,45]
	Inconsistent results on the antidepressant impact on BMD	Clinical Study	[46]
	Diosgenin enhances brain-derived serotonin to protect bone	Experimental Study	[47]

**Table 3:** Major bone-derived factors and their potential effects on depression.

Bone-Derived Factor	Primary Producing Cells	Mechanism of Action	Association with Depression
Osteocalcin (ucOC)	Osteoblasts	Activates GPR158 receptor and enhances serotonergic neurotransmission	Reduced levels in depressed patients
Osteopontin	Osteoblasts	Not fully elucidated	Influences depression pathogenesis
Extracellular Vesicles	Bone cells	Cross BBB → improve cognitive function in AD mice	Demonstrated in AD models; depression relevance pending
RANKL	Osteoblasts	Modulates neuroinflammatory responses	Anti-RANKL therapy ameliorates depressive behaviors
Lipocalin-2 (LCN2)	Osteoblasts/Osteocytes	Inhibits hippocampal neuroplasticity	Serum levels positively correlate with post-stroke depression risk

**Table 4:** Potential therapeutic strategies targeting the bone–brain axis and their functional characteristics.

Intervention Strategy	Molecular/Cellular Target	Effects on Depression	Effects on Bone	Development Stage
rTMS	Prefrontal cortex, motor cortex	Enhances emotional regulation and modulates limbic circuitry	Potential osteogenic effects and may elevate bone formation markers	Clinical validation
Combined aerobic-resistance exercise	Muscle–bone–brain axis	Reduces depressive symptoms and improves hippocampal neurogenesis	Increases bone mineral density and enhances bone microstructure	Clinically established
Osteocalcin analogs	GPR158 receptor	Potentiates serotonergic transmission and promotes antidepressant-like effects	Stimulates osteoblast activity and improves bone turnover balance	Preclinical development
Melatonin receptor agonists	Central/peripheral clocks	Restores sleep architecture and attenuates circadian mood fluctuations	Suppresses osteoclastogenesis and reduces nocturnal bone resorption	Clinical trials
LCN2-neutralizing antibodies	Choroid plexus LCN2 receptors	Normalizes tryptophan metabolism and restores 5-HT synthesis	Decreases bone loss and preserves trabecular architecture	Preclinical investigation

## Data Availability

No new data were created or analyzed in this study.
